# Neural regulation of the kidney function in rats with cisplatin induced renal failure

**DOI:** 10.3389/fphys.2015.00192

**Published:** 2015-06-30

**Authors:** Niamh E. Goulding, Edward J. Johns

**Affiliations:** Renal Research Laboratory, Department of Physiology, University College CorkCork, Ireland

**Keywords:** renal innervation, baroreflexes, chronic kidney disease, inflammation, renal sodium excretion

## Abstract

**Aim:** Chronic kidney disease (CKD) is often associated with a disturbed cardiovascular homeostasis. This investigation explored the role of the renal innervation in mediating deranged baroreflex control of renal sympathetic nerve activity (RSNA) and renal excretory function in cisplatin-induced renal failure.

**Methods:** Rats were either intact or bilaterally renally denervated 4 days prior to receiving cisplatin (5 mg/kg i.p.) and entered a chronic metabolic study for 8 days. At day 8, other groups of rats were prepared for acute measurement of RSNA or renal function with either intact or denervated kidneys.

**Results:** Following the cisplatin challenge, creatinine clearance was 50% lower while fractional sodium excretion and renal cortical and medullary TGF-β1 concentrations were 3–4 fold higher in both intact and renally denervated rats compared to control rats. In cisplatin-treated rats, the maximal gain of the high-pressure baroreflex curve was only 20% that of control rats, but following renal denervation not different from that of renally denervated control rats. Volume expansion reduced RSNA by 50% in control and in cisplatin-treated rats but only following bilateral renal denervation. The volume expansion mediated natriuresis/diuresis was absent in the cisplatin-treated rats but was normalized following renal denervation.

**Conclusions:** Cisplatin-induced renal injury impaired renal function and caused a sympatho-excitation with blunting of high and low pressure baroreflex regulation of RSNA, which was dependent on the renal innervation. It is suggested that in man with CKD there is a dysregulation of the neural control of the kidney mediated by its sensory innervation.

## Introduction

Chronic kidney disease (CKD) is often initiated as a consequence of structural deterioration within the renal vascular and tubular structures and impairs renal function which eventually impinges on cardiovascular homeostasis (Khawaja and Wilcox, 2011; Sobotka et al., 2011). Hypertension frequently develops in CKD patients, which not only causes further progressive damage to the kidneys, but is a major contributing factor to increased risk of cardiovascular events and mortality (Klag et al., 1996).

There is now compelling evidence that the sympathetic nervous system is over-activated in CKD. Plasma catecholamine levels have been shown to be increased in hemodialysis patients (Henrich et al., 1977). Moreover, patients with end stage renal disease have elevated muscle sympathetic nerve activity (MSNA) and that removal of the diseased kidneys, at or following transplantation of a functional kidney, decreased blood pressure, peripheral resistance, and the bursting rate in MSNA to normal values (Converse et al., 1992; Hausberg et al., 2002). Moreover, it has been reported that ablation of the renal nerves in patients with CKD, whilst not impacting on renal function itself not only delayed the deterioration in kidney function but also resulted in a chronic reduction in blood pressure (Ott et al., 2015).

It is not clear how the development of CKD may cause a sympatho-excitation as the disease progresses. Activation of the renal sympathetic nerves, that is the efferent innervation, can influence cardiovascular homeostasis by impacting on the regulation of extracellular fluid volume and hence blood pressure. This is due to the direct actions of the sympathetic nerves on renal resistance vessels, to increase vascular resistance and at the nephrons to stimulate tubular sodium and water reabsorption (Johns et al., 2011). Increased renal sympathetic nerve activity (RSNA) will also enhance the release of renin and the generation of angiotensin II which itself is not only a vasoconstrictor, but will also act directly on the proximal tubule to stimulate fluid reabsorption, and indirectly by increasing aldosterone production which causes sodium reabsorption at the distal tubule.

The kidney itself contains sensory nerves which have an important physiological role in the neural control of kidney function and may contribute to the deranged autonomic control in CKD (Kopp, 2015). Sensory nerves present in the renal pelvis appear to be sensitive chemo- and mechano-receptors which upon activation cause a renal sympatho-inhibition and a renal nerve dependent natriuresis at the contra-lateral kidney (Dibona and Rios, 1980). This is termed an inhibitory reno-renal reflex as it is likely to be involved in ensuring that excretion of a sodium and water load is distributed equitably between the two kidneys. There is evidence of an excitatory reno-renal reflex (Ditting et al., 2012; Johns, 2014) which elicits a sympatho-excitation. Early evidence by Katholi et al. (1983) using intrarenal adenosine administration in the dog and Smits and Brody (1984) and more recently Barry and Johns (2015) using intra-renal bradykinin infusion in the rat demonstrated acute increases in blood pressure and RSNA which was blocked in animals subjected to a bilateral renal denervation.

The recent debate and apparently conflicting findings on the role of the renal innervation in pathophysiological states in man, such as resistant hypertension, CKD and diabetes (Bhatt et al., 2014; Esler, 2014; Krum et al., 2014), has created uncertainty especially as the underlying physiological mechanisms are unclear. The hypothesis explored in this investigation was that injury to the kidney, which may induce inflammatory responses, would cause an activation of the renal sensory innervation leading to a renal sympatho-excitation and a dysregulation of baroreflexes and an inability to excrete a saline volume load which was mediated by the renal innervation. The model chosen was the cisplatin induced renal failure rat model in which the high and low pressure baroreflex regulation was tested by increasing and decreasing blood pressure and administering an acute saline volume load, respectively.

## Methods

Male Wistar rats, weighing 250–300 g were housed under a 12 h light/dark regime at 20 ± 3°C and 35% humidity. All procedures were approved by the Animal Experimentation Ethical Committee at National University Ireland, Cork and were performed in accordance with the European Community Directive 86/609/EC. All rats were maintained on a normal diet and tap water *ad libitum*.

### Cisplatin induced renal failure

Groups of rats were injected with either 5 mg/kg cisplatin (10 mg/ml, Hospira, Illinois, USA) in a volume of 6 ml/kg to induce renal injury, or the equivalent volume of saline (0.9% sodium chloride) intraperitoneally in the control groups of rats (Khan et al., 2007; Salman et al., 2011). Metabolic studies were performed over the subsequent 8 day period and acute studies were undertaken on day 8, post cisplatin administration.

### Metabolic studies

Rats were housed in metabolic cages for 8 days. Urine flow, sodium excretion, and water intake were measured over 24 h periods. Day 1 measurements represented basal values and were obtained prior to administration of either vehicle or cisplatin.

Bilateral renal denervation: Rats were anaesthetized (2–3% isoflurane in O_2_). Sequentially, left and right kidneys were exposed retroperitoneally, each renal artery was identified, stripped of its adventitia and then coated with a solution of 10% phenol in absolute ethanol for 1 min and then rinsed with saline. In control rats, the renal arteries were only exposed, but not manipulated further. The muscles and skin were sutured and an analgesic (carprofen 5 mg/kg, Pfizer Inc, USA) given subcutaneously. The animals were allowed 4 days to recover before cisplatin administration and entering the metabolic study.

Food was given *ad libitum* over the 8-day period. Urine samples and tail vein blood samples (0.4 ml) were taken on Days 1 and 8. The blood samples were centrifuged, the plasma was removed and frozen at −20°C. Day 1 and 8 samples of urine were also frozen at −20°C. The plasma and urine sodium concentrations were measured using Flame photometry (Corning, Halstead, Essex, UK); plasma and urine creatinine levels were measured (Quanticom Creatinine Assay DICT 500, Bioassay Systems, Hayward CA, USA) to allow estimation of the clearance of creatinine. Noradrenaline levels in plasma and urine were measured using an ELISA (LDN, Nordhorn, Germany). Noradrenaline excretion rates most likely reflect both filtration from plasma as well as renal production of the catecholamine and will be influenced by the ongoing level of glomerular filtration rate. In order to take account of the low filtration rate in the RF rats, noradrenaline excretion was calculated per unit filtrate (creatinine clearance) to attain a more direct measure of noradrenaline production. On day 8, the animals were killed with an overdose of anaesthetic (5 ml i.p. chloralose-urethane), the kidneys were excised, decapsulated, the cortices and medullae homogenized, and the supernatent frozen at −80°C for the later estimation of TGF-β1 (RnD Systems, Minneapolis, USA).

### Acute studies

General Surgical Preparation: Rats were anaesthetized using 1 ml i.p. of chloralose-urethane (16.5 and 250 mg/ml, respectively) and maintained under anaesthesia with further doses of 0.05 ml every 30 min. Rats were prepared for renal nerve recordings or renal functional measurements from the left kidney as described previously (Zhang et al., 1997; Huang and Johns, 1998). Briefly, cannulae were placed in the right femoral artery for measurement of mean arterial pressure (MAP) and right femoral vein for administration of sustaining saline and drugs. MAP and heart rate (Henrich et al.) were recorded using a pressure transducer (Spectromed, Oxnard, CA, USA) and an amplifier (Grass Instruments, Quincy, MA, USA). The renal nerves of the left kidney were dissected free and sealed onto multi-stranded stainless steel wire recording electrodes (Medwire Mt. Vernon, NY, USA) using dental glue (Klasse4Dental, Augsburg, Germany). Following surgical preparation, the rats were allowed to recover for 2 h before the experimental protocol began.

RSNA is the efferent nerve traffic passing from the spinal cord to the kidney whereas renal afferent, or sensory, nerve activity represents that arising within the kidney and passing to the CNS. In the present study, afferent nerve activity was not recorded as it was filtered out by the low and high pass filters in the amplifier. RSNA was verified by audio recognition and was amplified using an optically isolated amplifier (Grayden Electronics, Birmingham, UK) having a gain of 100 thousand with high and low pass filters set at 100 and 1000 Hz, respectively. MAP, HR, and RSNA were recorded using LabVIEW software (National Instruments, Austin, TX, USA), for offline analysis. At the end of the experiment the rats were killed with an overdose of anaesthetic and 20 min later, background RSNA was determined and this value taken from all other RSNA measurements. RSNA values for all rats were normalized to 100% at baseline levels.

In the acute studies, where left RSNA was recorded, bilateral renal denervation comprised exposing the right kidney and denervating it as described above. Thereafter, the nerves of the left kidney were mechanically occluded between the electrode and the kidney, just before application of the dental glue, to allow recording of RSNA only.

Low Pressure Baroreceptor Challenge: This comprised an acute saline volume expansion where an i.v. infusion of saline was administered at a rate of 0.25% of body weight per minute for 30 min. The MAP, HR, and RSNA responses to volume expansion were recorded continuously and averaged over 5 min periods for the duration of the 30 min protocol.

High Pressure Baroreceptor Challenge: High pressure baroreflex regulation of RSNA was evaluated using i.v. doses of phenylephrine and nitroprusside (10 μg in 0.2 ml of saline each) to increase and decrease MAP respectively, by approximately 50–60 mmHg. Each drug was infused at a rate of 0.05 ml per 10 s over a 40 s period. The order in which each drug was infused first was randomized throughout each experiment. A 30 min recovery period was allowed after the infusion of each drug, to enable RSNA to return to baseline value.

Baroreflex gain curves were generated by plotting the relationship between RSNA and MAP from offline-stored data. The voltage level of RSNA recorded in each animal is very much dependent on technical conditions, anatomical display, ease of dissecton and fatty tissue surrounding the nerve bundle and this creates large variability in the signal recorded. In order to decrease this variability, RSNA was normalized by taking the basal level recorded immediately prior to the administration of the vasopressor or vasodepressor agent as 100%. The resultant sigmoidal relationship was analyzed by means of the following 4 parameter logistic regression equation (Kent et al., 1972), using the software MATLAB (The Mathworks Inc, Cambridge, UK):

RSNA/HR=A4+A1{1+ exp[A2 ∗(MAP−A3)]}

where, A1 is the response range of HR and RSNA, A2 is the slope of the curve, A3 is the pressure at the midrange of the curve and A4 is the minimum response of HR and RSNA. A1–A4 values were determined for each rat and a mean value for A1–A4 calculated for each group.

The gain of the baroreflex control of HR and RSNA was calculated from the first derivative of the logistic equations as follows:

$$\begin{array}{l} Gain=-A1.A2.exp\;(A2\;(MAP-A3))/(1\\ \;+exp\;(A2\;(MAP-A3)){)}^{2} \end{array}$$

The maximum slope or gain (Gmax) was calculated at the midpoint (A3) from the 1st derivative of the logistic function:

$$G_{max}=-A1\;*A2/4$$

Renal Functional Protocol: Rats were anaesthetized and prepared as described above but following exposure of the left kidney, the ureter was cannulated for urine collection.

**Figure 1:**
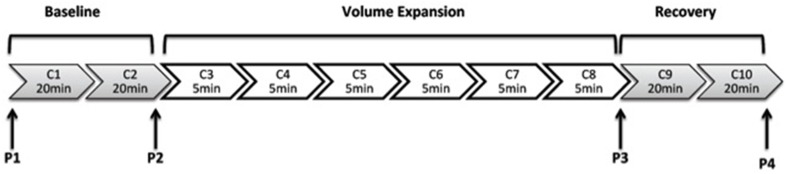
**This illustrates the protocol for urine collections for clearance (C) measurements**. C1, C2, C9, and C10 are of 20 min duration and C3–C8 are of 5 min duration.

Following surgical preparation, the rat was allowed to recover for 2 h before the experimental protocol began. The protocol (Figure 1) involved 10 clearance periods (C): two 20 min clearances to determine baseline values, followed by six 5 min collections during the saline volume expansion and then a further two 20 min clearances commencing 30 min after the end of the saline infusion. Plasma samples were collected into heparinized syringes from the arterial line at the four time points (P1–P4). The protocol of urine collections and plasma sampling are shown in Figure 1.

The metabolic study, acute RSNA and renal functional studies comprised the following groups of animals:

Control rats (*n* = 12) received a saline i.p. (6 ml/kg body weight)
RF rats (*n* = 12) were given 5 mg/kg of cisplatin i.p.
Bilaterally renally denervated (DNX) rats, control (*n* = 8)
Bilaterally renally denervated (DNX) RF rats (*n* = 8).

### Statistics

All statistical analyses were performed using Graphpad Prism Version 6.0c. Results are presented as means ± standard error of the mean (SEM). For all comparisons, statistical significance was taken at *P* < 0.05. When multiple groups were compared, a Two-Way analysis of variance (ANOVA) with Bonferonni's corrections for multiple comparisons were used, followed by Tukey's *post hoc* test. Paired and unpaired, two-tailed student's *t*-tests were employed when comparing two groups.

## Results

### Metabolic study

Table 1 shows that in the control rats, the fractional excretion of sodium (FENa), noradrenaline excretion and urine flow rate did not change from day 1 to 8. By contrast, following cisplatin administration in the RF rats, FENa and noradrenaline excretion were some three-fold higher on day 8 (both *P* < 0.05), although urine flow rate remained at the same level. Cisplatin given to the renally denervated rats (RF DNX) had no effect on FENa, noradrenaline excretion or urine flow rate at day 8 as the values were not significantly different from those recorded on day 1.

**Table 1:** **Metabolic data in all experimental groups of renal failure (RF), with (DNX) or without (INN) renal denervation, and control rats**.

	**Group**	**Day 1**	**Day 8**
Body Weight (g)	Control INN	272 ± 8	286 ± 7
	RF INN	268 ± 6	248 ± 12
	RF DNX	271 ± 5	281 ± 7^*^^#^
UNaV (μMh^−1^kg^−1^)	Control INN	157 ± 5	164 ± 7
	RF INN	157 ± 4	154 ± 6
	RF DNX	155 ± 5	159 ± 5
FE_Na_(%)	Control INN	0.9 ± 0.11	0.8 ± 0.10
	RF INN	0.7 ± 0.02^#^	2.5 ± 0.36^*^^#^
	RF DNX	1.12 ± 0.21	4.86 ± 0.22
NER (ngmL^−1^h^−1^)	Control INN	0.037 ± 0.006	0.024 ± 0.005
	RF INN	0.053 ± 0.005	0.189 ± 0.024^*^^#^
	RF DNX	0.045 ± 0.010	0.063 ± 0.013
GFR (mLmin^−1^kg^−1^)	Control INN	2.3 ± 0.2	2.0 ± 0.1
	RF INN	2.7 ± 0.3^#^	1.1 ± 0.2^*^^#^
	RF DNX	4.0 ± 0.2^*^	1.4 ± 0.5
UV (mLh^−1^kg^−1^)	Control INN	1.0 ± 0.1	0.5 ± 0.1
	RF INN	1.6 ± 0.3	1.8 ± 0.5^*^
	RF DNX	0.5 ± 0.1	0.6 ± 0.2

*Statistical analysis was performed using Two-Way ANOVA followed by Tukey's post hoc test. Data presented as mean SEM. (Control INN n = 12, RF and RF DNX, n = 8 per group). UNaV, absolute sodium excretion; FE_Na_, fractional excretion of sodium; NER, noradrenaline excretion rate; GFR, glomerular filtration rate (creatinine clearance); UV, urine flow rate*.

* *P < 0.05 vs. control INN*,

*#P < 0.05 vs. RF DNX*.

**Figure 2:**
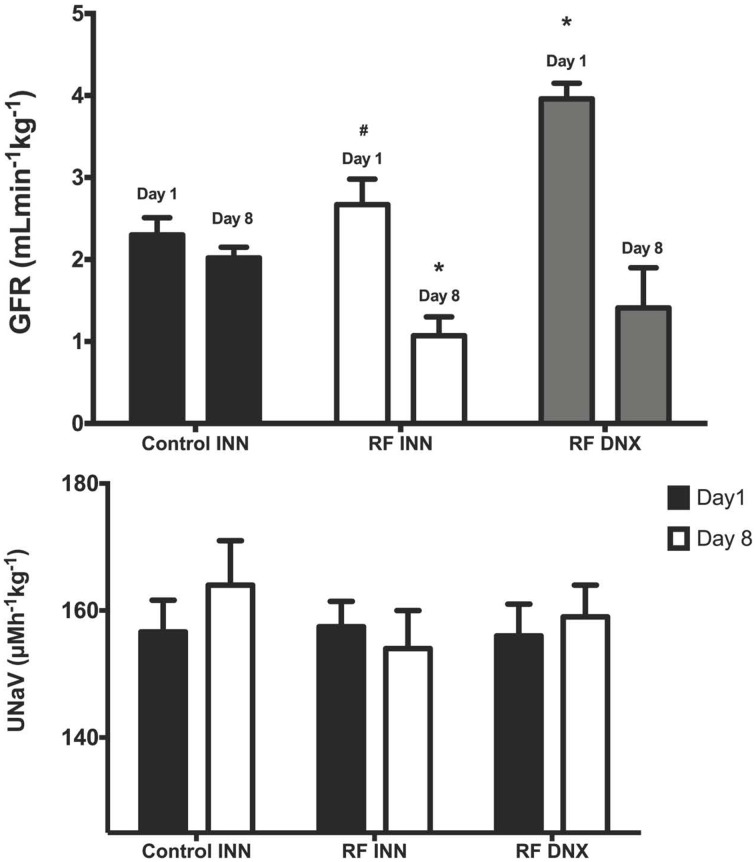
**Creatinine clearance (glomerular filtration rate, GFR), upper graph (A) and sodium excretion (Lower graph, B) for innervated (INN) control, renal failure (RF INN) and renally denervated RF (RF DNX) rats on day 1 and 8 of the metabolic study**. Statistical analysis was performed using Two-Way ANOVA followed by Tukey's *post-hoc* test. Data presented as mean SEM. (*n* = 12 for all groups). ^*^P < 0.05 vs. Control INN; ^#^*P* < 0.05 vs. RF DNX. *P*-value for interaction <0.005.

Creatinine clearance (Figure 2) remained at a stable unchanged value on day 8 in the control rats given vehicle on day 1. However, in the rats with an intact innervation but given cisplatin (RF) and those subject to prior renal denervation (RF DNX), there were significant reductions in creatinine clearance by day 8 of over 50% (*P* < 0.001). The day 1 value for creatinine clearance was significantly (*P* < 0.001) higher in the RF DNX compared to the control rats. There were no significant differences in absolute sodium excretion (UNaV) between any groups (Table 1 and Figure 2).

### TGF-β1

**Figure 3:**
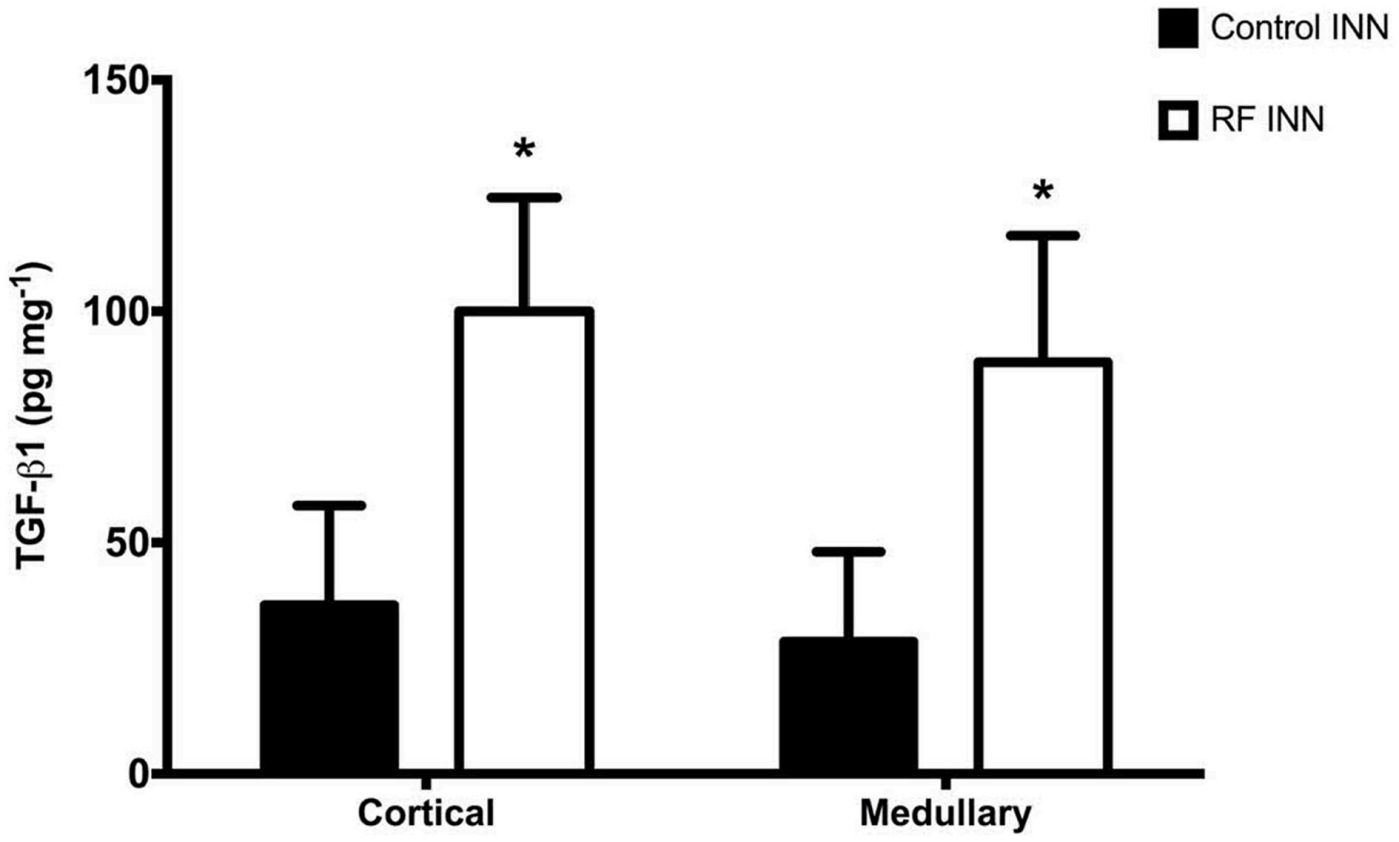
**This presents the renal cortical and medullary concentrations of TGF-β1 in control and RF groups of rats**. ^*^P < 0.001 vs. Control INN.

Renal cortical and medullary concentrations of TGF-β1 of kidneys taken from rats at day 8 were significantly (*P* < 0.0001) higher in the cisplatin treated than in control rats (Figure 3).

### Noradrenaline

**Figure 4:**
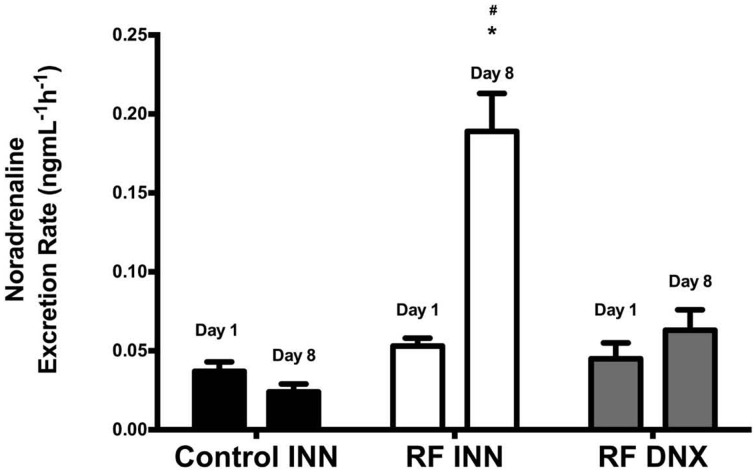
**This provides the fractional noradrenaline excretion rates (ng/mlGFR/h) for innervated control, innervated cisplatin treated (RF INN) and renally denervated renal failure (RF DNX) rats**. Statistical analysis was performed using Two-Way ANOVA followed by Tukey's *post hoc* test. Data presented as mean SEM. (*n* = 12 for all groups). ^*^P < 0.05 vs. Control INN ^#^*P* < 0.05 vs. RF DNX. *P*-value for interaction <0.0001.

Figure 4 shows that noradrenaline excretion rates did not change significantly in control rats from day 1 to 8. However, noradrenaline excretion increased significantly (*P* < 0.0001) from baseline levels at day 1 by approximately four fold by day 8 in the RF group of rats, whereas in the RF DNX group, noradrenaline excretion at day 8 was not significantly different from that measured at day 1.

### Acute studies

**Figure 5:**
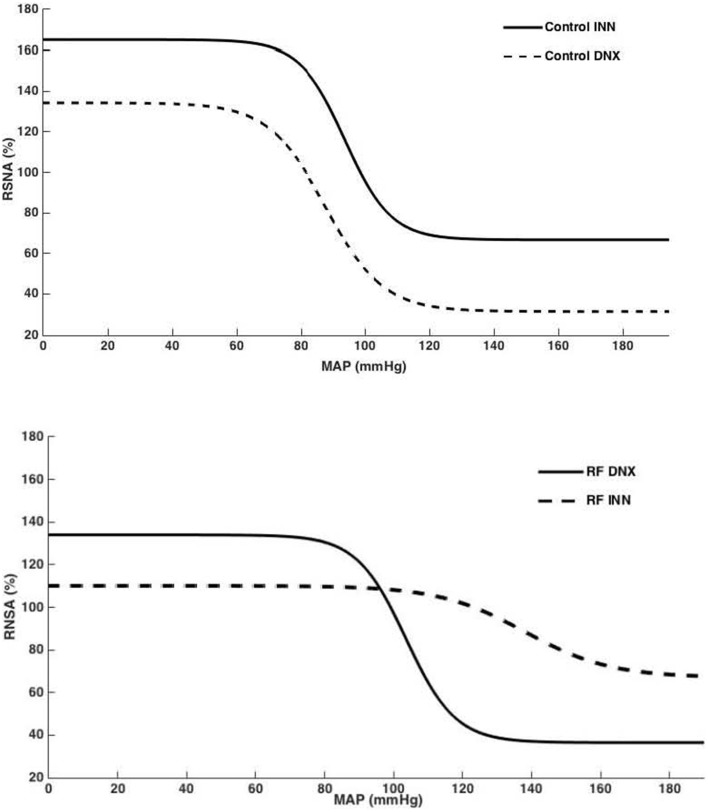
**Baroreflex curves for RSNA (%), generated from mean A1–A4 values for control innervated (control INN) and denervated (control DNX) and renal failure (RF) innervated (RF INN) and denervated (RF DNX) groups**.

**Figure 6:**
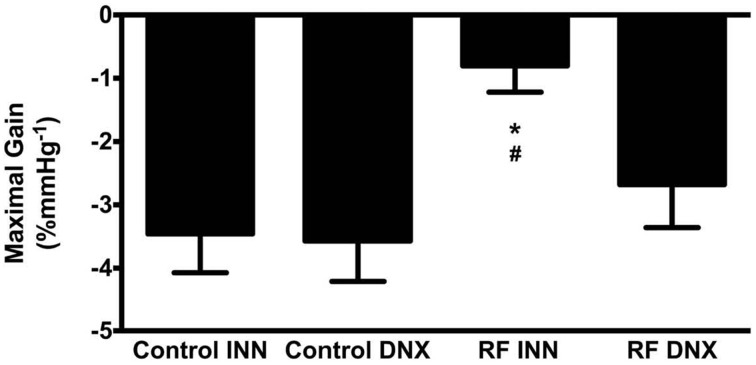
**This illustrates the maximal gain of the RSNA baroreflex curve for intact control (Control INN), control renally denervated rats (control DNX), cisplatin treated renal failure (RF) and renally denervated renal failure (RF DNX) groups of rats**. Statistical analysis was performed using Two-Way ANOVA followed by Tukey's *post hoc* test. Data presented as mean SEM. (*n* = 12 for all groups). ^*^P < 0.05 vs. Control INN; ^#^*P* < 0.05 vs. RF DNX.

High Pressure Baroreflex challenge: It can be seen from Table 2 that baseline values of HR and MAP were not different in all groups of rats. Basal integrated RSNA was significantly (*P* < 0.005) higher in the RF group when compared with all other groups. In the RF animals, both the range (A1) and the slope (A2) of the gain curve were significantly lower and mid-point blood pressure (A3) higher (*P* < 0.05, <0.01, and <0.05, respectively) compared to those of the control rats although and the minimum point to which RSNA could be driven (A4) were no different. Renal denervation of the control rats had little effect on the values of A1, A2, or A3, although A4 was reduced. However, following renal denervation in the RF rats the values of A1, A2, A3, and A4 could not be distinguished statistically from those of the control DNX rats. These findings are illustrated as full baroreceptor gain curves in Figure 5. Figure 6 presents the maximal gain of the RSNA baroreflex curve for all groups and it was significantly (*P* < 0.05) lower in the RF compared to the control rats. Furthermore, renal denervation of the RF rats (RF DNX) resulted in maximal gain values for RSNA which were no different from either the control or control DNX groups of rats.

**Table 2:** **Baseline mean arterial pressure (MAP), heart rate (HR) and renal sympathetic nerve activity (RSNA) and baroreflex gain curve parameters**.

	**Control INN**	**RF INN**	**Control DNX**	**RF DNX**
Baseline HR (beats min^−1^)	346 ± 27	347 ± 13	358 ± 18	361 ± 20
Baseline MAP (mmHg)	94 ± 7	83 ± 4	72 ± 5	78 ± 3
Baseline RSNA (mVs^−1^)	7 ± 3	29 ± 6^*^^#^	4 ± 1	2 ± 0.3
A1 (% RSNA)	98 ± 19	43 ± 15^*^	103 ± 8	98 ± 15
A2 (% RSNA mmHg^−1^)	0.14 ± 0.01	0.07 ± 0.02^*^^#^	0.11 ± 0.03	0.14 ± 0.02
A3 (mmHg)	94 ± 17	138 ± 17^*^^#^	88 ± 8	104 ± 6
A4 (% RSNA)	68 ± 23	68 ± 9^#^	31 ± 2	37 ± 7

*A1, the range of the curve; A2, the slope or sensitivity of the curve; A3, the mid-point blood pressure; and A4, the minimum point to which the RSNA could be driven in all experimental groups. Renal failure (RF) and control rats, with (DNX) or without (INN) renal denervation. Statistical analysis was performed using Two-Way ANOVA followed by Tukey's post hoc test. Data presented as mean SEM. (n = 12 for control and n = 8 for RF groups)*.

* P < 0.05 vs. control INN; *P < 0.005 vs. control

# *P < 0.05 vs. RF DNX*.

**Figure 7:**
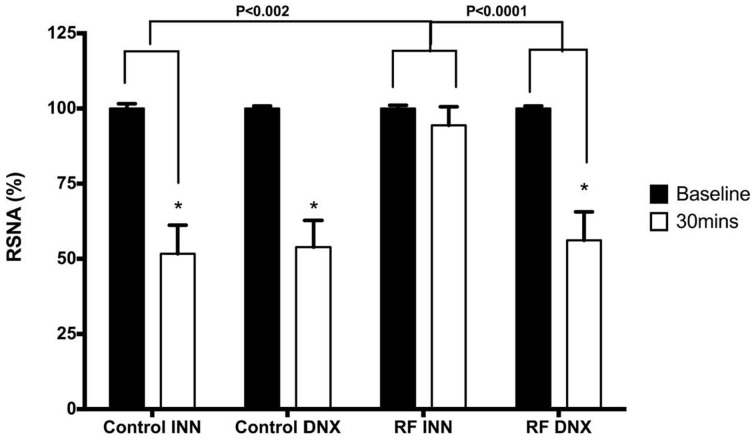
**This compares the changes in renal sympathetic nerve activity (RSNA) in response to the saline volume expansion at baseline (over the 5 min prior to the start of infusion) and over the last 5 min of the infusion (30 min) in intact control (Control INN), control renally denervated rats (control DNX), intact cisplatin treated renal failure (RF) and renally denervated renal failure (RF DNX) groups of rats**. Statistical analysis was performed using Two-Way ANOVA followed by Tukey's *post hoc* test. Data presented as mean SEM. (*n* = 12 for all groups). ^*^P < 0.001 against baseline. *P*-value for interaction <0.005.

Low pressure baroreflex challenge: The infusion of the acute saline load (Figure 7) significantly decreased RSNA by approximately 50% after 30 min in both the intact control rats and those subjected to renal denervation (both *P* < 0.05). By contrast, in the RF rats, the acute saline load did not change RSNA, which was a response significantly (*P* < 0.002) different from that obtained in the control rats. However, after prior renal denervation of the RF rats, the 30 min of saline infusion decreased RSNA to the same degree as that obtained in the control and control DNX groups of rats, and to a significantly (*P* < 0.001) greater degree than that obtained in the intact RF group (Figure 7).

**Figure 8:**
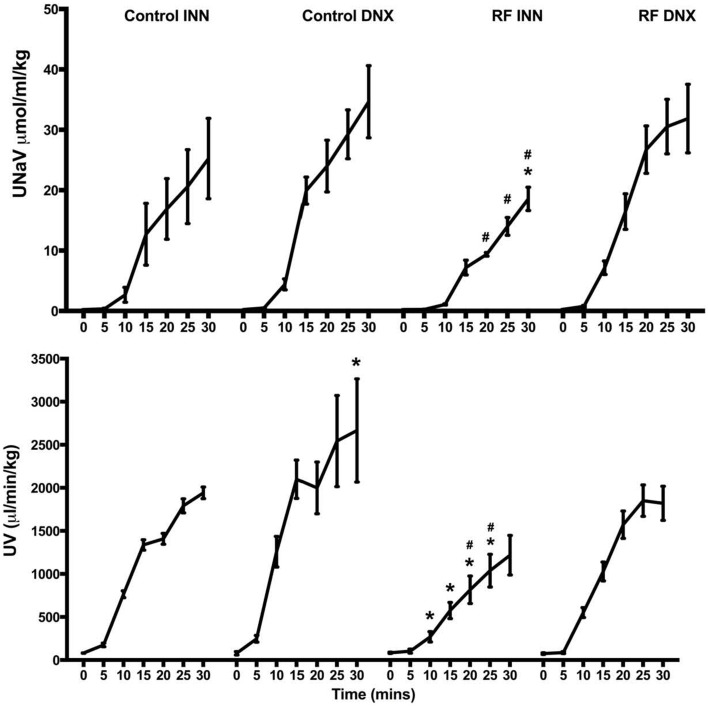
**This presents the values of urine flow (UV) and sodium excretion (UNaV) for each of the 5 min periods during the 30 min of saline volume expansion in control, control renally denervated rats (control DNX), cisplatin treated renal failure (RF) and renally denervated renal failure (RF DNX) groups of rats**. Statistical analysis was performed using Two-Way ANOVA followed by Tukey's *post hoc* test. Data presented as mean SEM. (*n* = 12 for all groups). ^*^P < 0.01 vs. control ^#^*P* < 0.05 vs. RF DNX. *P*-value for interaction is <0.001.

The excretory responses of all groups of rats control and renal failure rats over the course of the volume expansion are presented in Figure 8. The acute volume load significantly increased UV and UNaV from 81.1 ± 2.0 to 1941.3 ± 67.5 μl/min/kg and 0.22 ± 0.01 to 25.24 ± 6.66 μmol/ml/kg, respectively in the control group and to a significantly greater extent in the control rats subjected to renal denervation, from 78.3 ± 19.1 to 2665.5 ± 598.5 μl/min/kg and 0.23 ± 0.01 to 34.65 ± 5.97 μmol/ml/kg, respectively (*P* < 0.01). The magnitude of increase in UV was significantly (*P* < 0.001) larger in the control compared to the RF group. The magnitudes of increase in UV and UNaV in response to the saline volume expansion were significantly blunted in the RF rats (both *P* < 0.001) but not in the RF DNX rats in which the increases in UV and UNaV were similar to those of the control rats.

## Discussion

The role of the renal sensory innervation in the regulation of autonomic and renal function remains enigmatic. There has recently been renewed interest in the contribution of the renal innervation in specific patient groups; those with resistant hypertension (Krum et al., 2014), chronic renal disease (Ott et al., 2015) and diabetes and obesity (Mahfoud et al., 2011). However, the apparent failure of the Symplicity HTN3 trial (Bhatt et al., 2014) has called into question the contribution of the renal nerves in these patho-physiological states and indicates that further study is necessary.

The present investigation set out to explore how renal failure following injury to the kidney with cisplatin impacted on the neural regulation of kidney function. There were two novel findings. Firstly, that the cisplatin induced renal injury was associated with a sympatho-excitation, as shown by the increase in fractional noradrenaline excretion, and that it was an effect dependent on an intact renal innervation. Secondly, that the deranged high and low pressure baroreflex regulation of RSNA following the renal injury with cisplatin resulted in a blunted ability to excrete a sodium load and, moreover, was dependent on the renal innervation as the natriuretic and diuretic responses were normalized following removal of the renal innervation. These findings reveal that the injured and failing kidney seems to elicit an activation of the renal sensory innervation which blunts sympathetic control preventing the dynamic regulation of renal excretory function necessary for the regulation of extracellular fluid volume and hence the chronic level at which blood pressure is set.

The administration of a single dose of cisplain caused a marked reduction in creatinine clearance indicative of decreased glomerular filtration comparable to that reported earlier using this model (Khan et al., 2007). This likely reflects damage caused by cisplatin to the proximal epithelial cells, which slough off and generate hyaline casts that in turn block the tubules decreasing filtration. Indeed, injury to the kidney is supported by the observation that concentrations of TGF-β1, a biomarker of renal damage and fibrosis (Bottinger, 2007), was increased to high levels in both the renal cortex and medulla by day 8. One of the consequences of cisplatin induced renal injury will be an activation of the renin-angiotensin-aldosterone system. Any increase in angiotensin II production will contribute to the deterioration in renal function in two ways, directly by causing constriction of the renal resistance vessels, and indirectly via a facilitation of noradrenaline release from the varicosities of the postganglionic sympathetic fibers at the neuroeffector junctions. Although the cisplatin injury decreased creatinine clearance, absolute sodium excretion was maintained at levels comparable to those of the control rats, but this most likely reflects an adjustment of fluid handling along the later portions of the nephrons. The end result was that by day 8, fractional sodium excretion, was markedly elevated in the cisplatin treated rats, which indicated that there was decreased reabsorption of fluid along the nephron as a greater proportion of the filtered load was excreted. It was clear that in the renal failure group subjected to the renal denervation, following the cisplatin challenge there was a similarly elevated fractional sodium excretion by day 8 suggesting that the renal sympathetic innervation was playing a minor role in the regulation of basal fluid excretion under these conditions.

A primary aim of the investigation was to determine whether in the cisplatin model of renal injury there was an excitation of the sympathetic nervous system. To this end, the urinary excretion of noradrenaline was evaluated. Fractional noradrenaline excretion was evaluated even though this was a less reliable indicator of sympathetic nerve activity in the kidney than measurement of noradrenaline content, but it did have the advantage of allowing repeated measures in the same animals. The first novel finding was that fractional noradrenaline excretion was markedly elevated by day 8, indicative of an increase in sympathetic activity. It is likely that there are two sources of noradrenaline in the kidney, that filtered from the plasma, and that released at the neuroeffector junctions of the renal sympathetic innervation. Clearly, there is a reduced filtered load of both fluid and noradrenaline in the injured kidney but if this was taken into account, by calculating fractional noradrenaline excretion, then it became evident that that there was a large increase in noradrenaline excreted by the kidney. This conclusion was supported by the observation that in the animals subjected to the bilateral renal denervation, there was no change in fractional noradrenaline excretion on day 8, compared with baseline, following the cisplatin injection even though there was a comparable reduction in glomerular filtration rate. A limitation of the present study was that a group of control rats subjected to renal denervation were not studied but there was a low probability that any major responses in renal function would have occurred over the 8 day period of study. Interestingly, the level of RSNA recorded from the multifiber nerve recordings in the acute studies was higher in the renal failure model compared with the control rats. However, such a direct comparison of this nature is not valid because there are technical challenges which impact on the ability to get consistent nerve recordings between animals because of differing anatomical displays, the ability to clear fatty tissue from the nerve bundle and the number of nerve bundles that can be placed on the electrodes. Together, these findings imply that there is a sympatho-excitation following the cisplatin induced renal failure but perhaps more importantly, they suggest that it is the kidney which is the source of the sensory information passing into the central nervous system. Moreover, these observations closely reflect the reports in chronic renal disease in man where an elevated level of sympathetic activity has been reported (Converse et al., 1992; Hausberg et al., 2002).

The second major objective of the investigation was to determine how the cisplatin induced renal injury disrupted the baroreflex regulation of RSNA and renal nerve dependent excretory function. It was evident that there were marked alterations in the baroreflex gain curves in the renal failure rats. Perhaps surprising was the elevation in mid-point blood pressure (A3) in renal failure at a time when basal blood pressure was similar to that of the control rats. The reasons are unclear but one possibility is that basal blood pressure is influenced by the level of anaesthesia whereas the baroreflex gives a better reflection of the overall regulatory mechanisms that determine the level at which blood pressure is set. The maximal gain of the high pressure baroreflex was very much depressed in the renal failure rats demonstrating that autonomic control was blunted. Importantly, bilateral renal denervation restored the maximal gain of the baroreflex to normal values indicating that a neural signal was originating from the kidneys under these circumstances. These findings support an earlier report in renal failure in rats (Khan et al., 2014) evaluating slightly different characteristics of the baroreflex curves but nevertheless also demonstrate major dysfunction in baroreflex regulation in renal failure. Nonetheless, injury to the kidney elicits an inappropriate neural signal which, within the CNS, blunts normal high pressure baroreflex regulation of at least one major organ, the kidney, and could seriously impair cardiovascular homeostasis.

Challenging the cardiopulmonary reflex using an acute saline volume expansion caused a prompt renal sympatho-inhibition, which was a response absent in the renal failure animals but that could be restored by prior renal denervation. These observations support those of Khan et al. (2014) and reinforce the concept that inappropriate sensory information arising from the injured kidneys impairs the normal operation of the cardiopulmonary reflex. The second important novel observation was that the ability to increase sodium and water excretion in response to the volume expansion was very much attenuated but could be restored if the influence of the renal nerves was removed. Two interesting points arise from this observation. Firstly, that part of the inability to excrete the saline load in the renal failure rats could be due to the increased RSNA which, via the direct action of the nerves on proximal tubular fluid reabsorption, would cause a relative fluid retention. Secondly, the restoration of the excretory responses in the renal failure rats following renal denervation was compatible with an inappropriate sensory signal arising from the injured kidneys which was both causing an elevated RSNA as well as blunting the normal renal sympatho-inhibitory response to a volume expansion.

This investigation set out to examine how injury to the kidney, induced by cisplatin, caused a derangement of the reflex regulation of RSNA and the neural regulation of kidney excretory function. There is good evidence that in experimental models and man CKD is associated with a sympatho-excitation that may be due to the intra-renal generation of inflammatory mediators (Campese and Kogosov, 1995; Campese et al., 2011; Koeners et al., 2014). It was apparent in the present study that cisplatin induced renal failure was associated with an increased noradrenaline excretion consistent with a sympatho-excitation. There was also a marked attenuation of both the high and low pressure baroreflex regulation of RSNA and in terms of function, prevented the volume expansion mediated natriuresis and diuresis. Derangement of these reflexes means that the dynamic handling of sodium and water during normal everyday activity is lost which will seriously impact on cardiovascular homeostasis. Importantly, these dysfunctions appear dependent on the renal innervation as they are normalized when the kidneys are denervated. The question arises as to how an inappropriate sensory signal is generated within the kidneys under these conditions. In this renal failure model, an inflammatory response takes place as expressed by the increase in TGF-β1 concentrations within the kidney. One significant pro-inflammatory mediator within the kidney is bradykinin which is a key mediator of increased sensory nerve activity (Kopp, 2015) and recently it has been reported that intra-renal bradykinin infusion can increase RSNA, but not if the infused kidney is denervated (Barry and Johns, 2015). It may well be that an inflammatory response induced by renal injury is responsible for the deranged neural control of the kidney as renal disease develops.

### Conflict of interest statement

The authors declare that the research was conducted in the absence of any commercial or financial relationships that could be construed as a potential conflict of interest.
